# Savoring Satiety: An Exploratory Analysis of the Neural Correlates of Sensory-Specific Satiety

**DOI:** 10.3390/nu17203229

**Published:** 2025-10-15

**Authors:** Joe J. Simon, Tim Müller, Fabian Schöner, Martin Bendszus, Hans-Christoph Friederich

**Affiliations:** 1 Department of General Internal Medicine and Psychosomatics, University Hospital Heidelberg, 69120 Heidelberg, Germany; t.mueller.hd.94@gmail.com (T.M.); fabian.schoener@med.uni-heidelberg.de (F.S.); hans-christoph.friederich@med.uni-heidelberg.de (H.-C.F.); 2 German Centre for Mental Health, Partner Site Heidelberg/Mannheim/Ulm, 68159 Heidelberg, Germany; 3 Department of Neuroradiology, University Hospital of Heidelberg, 69120 Heidelberg, Germany; martin.bendszus@med.uni-heidelberg.de

**Keywords:** FMRI, sensory-specific satiety, food reward, BMI, neural reward processing, neural gustatory processing

## Abstract

**Background/Objectives**: Sensory-specific satiety (SSS) refers to the decrease in pleasantness of a food after repeated consumption, while other foods remain appealing. Despite its significance in hedonic food perception, the underlying mechanisms of SSS remain poorly understood. This study aimed to investigate the neurobiological basis of SSS and its relationship with body weight and hedonic food perception. **Methods**: Twenty-three healthy individuals with varying body weights underwent functional magnetic resonance imaging during a novel gustatory stimulation procedure. SSS was induced by repeated exposure to glucose, during which the hedonic perception of a neutral stimulus increased. **Results**: We found that SSS was associated with a network of brain regions related to reward and taste processing, including the lateral orbitofrontal cortex. Increased activation in the medial prefrontal cortex was related to both the expectation and receipt of a neutral stimulus with increased hedonic value during SSS. Finally, higher body weight was related to decreased activation in the medial prefrontal cortex, whereas an increased tendency for food craving was associated with increased activation of the lateral orbitofrontal cortex during SSS. **Conclusions**: Our results extend previous findings of an orbitofrontal-cortex-mediated shift in hedonic perception of food during SSS and show that the medial prefrontal cortex plays a crucial role in reward value modulation during SSS. Furthermore, our results indicate that increased BMI and trait food craving are associated with altered reward processing during SSS. Taken together, our results provide new insights into the neural mechanisms underlying changes in hedonic food perception during SSS.

## 1. Introduction

Food intake is not only driven by the need for energy and nutrients but is also influenced by various other factors, some of which are driven purely by pleasure. Amongst those, the phenomenon of sensory-specific satiety (SSS) has been found to directly impact the quantity of food consumed [1]. SSS occurs when the pleasantness of a consumed food decreases compared to that of uneaten foods [2]. The repeated consumption of the same food during a meal causes a habituation effect, irrespective of overall satiety [3], where the sensory attributes of the food eaten become less pleasurable than others. It has been suggested that SSS evolved as a mechanism to promote dietary variety [4] and has been found to play an important role in regulating food intake and satiety [5,6].

Since reduced SSS may lead to overeating by enabling larger meal sizes or quicker return to eating [7], previous studies have investigated its role in the development and maintenance of obesity. Interventional studies have attempted to induce long-term SSS by employing dietary interventions with reduced food variety but observed inconclusive effects on long-term weight loss [8,9,10]. So far, there is inconclusive evidence for impaired SSS in obesity. Animal models have shown intact SSS in obesity [11], and most studies investigating SSS in individuals with obesity have not observed reduced or increased SSS [12,13,14]. However, one study did find reduced SSS in participants with obesity [15]. Accordingly, a possible mechanistic relationship between impaired SSS and obesity remains unclear. Furthermore, research comparing pre- and post-COVID populations suggests that infection may have lasting effects on hormonal appetite regulation [16], together with disrupted olfactory and gustatory processing and altered reward-related neural circuits [17,18,19]. These findings indicate that satiety and food reward mechanisms may be shaped not only by metabolic status but also by broader public health contexts.

An extended network of mesocorticolimbic brain regions has been associated with the processing of hedonic and sensory properties of food, including areas in the bilateral insula, thalamus, and nucleus caudatus, as well as the orbitofrontal and anterior cingulate cortex [20,21,22]. However, studies investigating the neurobiological basis of SSS are scarce, and only a few studies have examined neural processing during SSS. Orosensory stimulation during food intake affects satiation by direct signaling from the brainstem to higher cortical regions involved in taste and reward [23]. Accordingly, previous studies found that an SSS-induced decrease in the pleasantness of food is related to a reduction of dopaminergic-mediated activity in the orbitofrontal cortex (OFC; [4,24]) and reduced neural processing related to sensory perception [25]. The orbitofrontal cortex (OFC) plays a central role in integrating sensory inputs from food with hunger and satiety signals, shaping how pleasurable a food is and whether it stimulates appetite [26,27]. It has, therefore, been hypothesized that SSS is encoded in the OFC, where neurons gradually decrease their responses to specific foods via orosensory stimulation [28]. For example, single-unit recordings in primates show that neurons in the lateral orbitofrontal cortex decrease their response selectively to the taste of a food that has been eaten to satiety while maintaining responses to other foods [29,30]. Complementary human fMRI studies demonstrate that the orbitofrontal cortex exhibits reduced activation to the smell or taste of a satiated food, an effect not observed for non-satiated alternatives [24,31].

However, a recent study assessed neural processing during repeated exposure to a highly palatable food taste (i.e., chocolate milkshake) and found conflicting results. The authors observed that, over the course of exposure, activation in brain regions associated with reward, gustatory processing, and executive functioning increased during both the processing of taste-signalling cues and the administration of taste [32]. Since they failed to observe a reduction of activation in the OFC, the authors suggested that the small volume of liquid administered may have been insufficient to trigger SSS and related OFC activation, instead resulting in an increased somatosensory response over time [32].

Taken together, previous findings highlight the need for further research into the neural basis of sensory-specific satiety. Using a novel gustatory fMRI paradigm with glucose and water, the present study investigated changes in hedonic perception during both anticipation and receipt of taste. Water was chosen as a neutral zero-energy stimulus, providing a baseline that allows context-dependent changes in pleasantness to be more clearly attributed to SSS. We expected that repeated glucose exposure would decrease the pleasantness of glucose while increasing the hedonic value of water, with corresponding neural changes in reward-related brain regions. We further hypothesized that body mass index (BMI) and food craving would modulate these neural responses.

## 2. Materials and Methods

### 2.1. Study Design and Test Group

Twenty-five healthy participants were recruited using flyer advertisements. All participants had normal or corrected-to-normal vision, were right-handed, and were screened for mental disorders via the Structured Clinical Interview for DSM-IV [33]. We recruited participants from a wide range of body weights (lowest BMI = 18.4 kg/m^2^, highest BMI = 32.2 kg/m^2^). Out of the 23 participants, 2 had a BMI below 20, and 1 had a BMI above 30. Additional exclusion criteria were pregnancy, magnetic implants, mental disorders (including lifetime occurrence of eating disorders), smoking, and claustrophobia. Participant characteristics are given in Table 1. Two participants had to be excluded due to excessive head movement during scanning (>4 mm translation or >±3° of rotation), resulting in a final sample size of 23. We employed the Trait and State Food Cravings Questionnaires to assess general (trait) and momentary (state) susceptibility to food cravings (FCQ-T and FCQ-S, [34]) and the Dutch Eating Behavior Questionnaire (DEBQ; [35,36]) to assess eating styles, including restrained, emotional, and external eating. The medical ethics committee of the Medical Faculty Heidelberg at the Ruprecht-Karls-University in Heidelberg, Germany, approved this study (protocol number S-656/2019), and written informed consent was obtained from all participants. The study was conducted in accordance with the Declaration of Helsinki.

### 2.2. Procedure

All participants underwent one fMRI session, which took place at lunchtime (between 12:00 and 13:30 h for all participants). Participants were asked to skip breakfast to achieve a fasting period of approximately 12 h. Each study visit began with the screening for mental disorders (see above), and participants were asked to fill out the questionnaires at home shortly before the study. Before entering the scanner, the experimental procedures as well as the task were explained to participants.

Once inside the scanner, participants underwent a gustatory stimulation paradigm. Taste stimuli were delivered into the mouth using a gustometer. The gustometer consists of 3 syringe pumps (Aladdin Pump, World Precision Instruments, Sarasota, FL, USA), equipped with 50 mL glass syringes (Fortuna Optima, Poulten & Graf GmbH, Wertheim, Germany) connected to Tygon^®^ beverage tubing (Saint-Gobain Performance Plastics, Aubervilliers, France). The tube lines converge to a single manifold fixed to the head coil, designed to obtain a reproducible delivery of flavored solutions onto the subject’s tongue based on the design used in previous studies [37,38]. To avoid backflow, syringe pumps were programmed to function at a flow rate of 10 mL/h. Stimuli were presented and responses recorded using Presentation V18 (Neurobehavioral Systems, Berkeley, CA, USA, RRID:SCR_002521).

**Figure 1:**
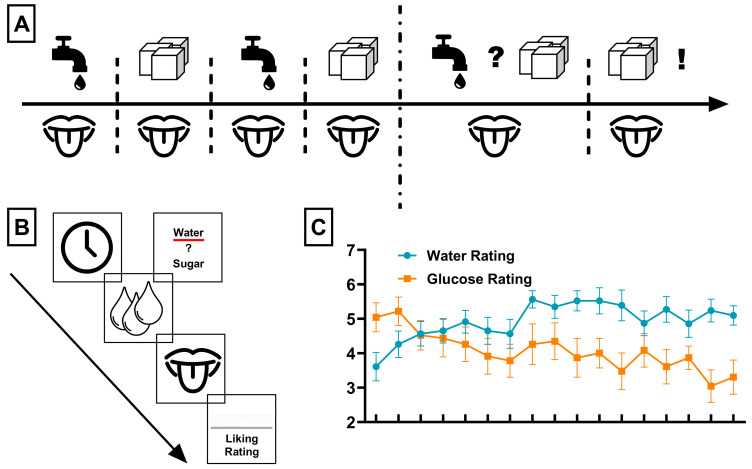
(**A**) Schematic outline of the gustatory stimulation task, including the stimuli presented during the experiment. During the first part of the task, participants passively received an infusion of either water or glucose. During the second part of the task, participants were able to choose between water or glucose but intermittently received passive infusions of glucose. (**B**) Structure of a single trial of the gustatory stimulation task. Each trial began with a cue indicating either the type of liquid to be infused (faucet symbol for water or sugar cubes for glucose) or, during the second part of the task, the ability to choose between the infusion of water and glucose. The liquid was then administered (indicated by the droplet symbol), and participants were then instructed to “taste” the liquid (indicated by the mouth-and-tongue symbol). At the end of each trial, participants were asked to rate the liking of the respective liquid. (**C**) Changes in liking ratings of water and glucose over time. Water and glucose ratings significantly increased and decreased over time, respectively (both linear regressions; *p*s < 0.005).

Participants first viewed a visual cue for 2 s, informing them of the incoming taste solution. Then, a total of 1 mL of taste solution (containing either a 10% glucose solution, a 20% glucose solution, or plain water) was delivered at a constant rate for 9 s. Subsequently, a swallow cue was displayed for 3 s after drink delivery, instructing participants to swallow the taste solution. Following this, participants rated the “liking” of the solution using a visual analog scale (VAS) for 6 s. Ratings were made on-screen using a horizontal visual analog scale ranging from not at all pleasant to very pleasant (on a 9-point Likert scale). Participants moved an on-screen cursor using the left and right buttons of a three-button response device and confirmed their rating with the middle button. At the end of each trial, 1 mL of water was delivered for 6 s, followed by a 3 s swallowing period to remove residual taste in the mouth. Each trial had a total duration of 30 s and was followed by a jittered inter-trial interval ranging from 3 to 5 s with a mean duration of 4 s. After every 5 trials, a 15 s pause was included. In the first half, pauses occurred after trials 5, 10, and 15; in the second half, after trials 5, 10, 15, and 20. During the first half of the task (19 trials, ~10 min), participants were presented with a 10% glucose solution and water in an alternating fashion. Two different glucose concentrations were used in order to prevent early habituation to a single intensity and because the higher concentration produces a more intense sweet taste, which facilitates the development of sensory-specific satiety over time. During the second half of the task (25 trials, ~15 min), trials alternated between choice events and fixed glucose deliveries, beginning with a choice event. There were 13 choice events and 12 fixed deliveries. Fixed deliveries alternated between 10% and 20% glucose, and there were six of each. In choice events, the cue announced a choice, and participants selected water or 10% glucose. This choice structure was implemented to bias selections toward water in the later phase of the task, thereby enabling us to assess whether repeated glucose exposure produced the anticipated revaluation effect on a neutral stimulus. In fixed deliveries, the cue announced the upcoming glucose concentration. Figure 1A gives the general outline of the task, and Figure 1B displays the structure of a single trial.

### 2.3. fMRI Acquisition

Functional imaging was performed using a Prisma Fit 3-Tesla whole-body MRI scanner (Siemens Medical Solutions, Erlangen, Germany) equipped with a standard 32-channel head coil. Thirty oblique interleaved slices with no interslice gap were acquired parallel to the AC-PC axis using a T2*-sensitive single-shot EPI sequence with the following parameters: TR = 2250 ms, TE = 30 ms, flip angle = 80°, and field of view = 192 × 192 mm with a matrix size of 64 × 64 and an in-plane resolution of 3 × 3 × 3 mm. The gustatory stimulation task was performed during one continuous run with 640 scans (for a total duration of 24 min). For anatomical reference, high-resolution T1 MPRAGE anatomical images were collected (176 slices, voxel size = 1 × 1 × 1 mm, TR = 1900 ms, TE = 2.26 ms, flip angle = 9°, field of view = 25.6 × 25.6 cm).

### 2.4. fMRI Analysis

Pre-processing and statistical analyses of fMRI data were performed using MATLAB, version R2024b (RRID:SCR_001622) and the Statistical Parametric Mapping toolbox (SPM12, Wellcome Department of Cognitive Neurology, Institute of Neurology, London, UK, RRID:SCR_007037). The first four volumes of each scan were excluded to account for magnetic field equilibration. Functional images were then corrected for differences in slice acquisition timing and realigned, allowing a maximum motion of ±4 mm translation and ±3° rotation across the entire experiment. Images were then unwarped to correct for artifacts caused by susceptibility-by-movement interactions. Individual T1 images were coregistered with the mean functional images and subsequently segmented. Structural and functional images were normalized to standard anatomical MNI space using the transformation parameters obtained from the segmentation, resulting in a voxel size of 3 mm^3^ for functional images. Furthermore, functional images were smoothed with an 8 mm full-width at half-maximum isotropic Gaussian kernel.

*First-level analysis.* BOLD activity related to individual events was modeled using a general linear model. The visual cue indication pending taste delivery, the phase of taste delivery, and swallowing were modeled as regressors of interest for each taste solution. The “liking” rating of each taste, rinsing following taste delivery, and inter-trial interval were included as additional regressors of no interest. A 128 s high-pass filter was included in the model to remove low-frequency noise and signal drift, and an autoregressive model was used to account for serial correlations in time series.

*Anticipation contrasts.* We assessed changes in the hedonic value of taste stimuli during anticipation by comparing visual cues for water and glucose before and during sensory-specific satiety (SSS). Choice events (when participants were able to select their preferred liquid) were compared to fixed events during the second half of the task.

*Infusion contrasts.* To examine neural processing during taste delivery, we compared passive infusions of water and glucose before SSS, and again during SSS. We also contrasted glucose before versus during SSS and water before versus during SSS to capture SSS-related changes in hedonic valuation.

This resulted in the following contrasts of interest:

(a) Choice of water during SSS compared to passive receipt of water before SSS (=influence of sensory-specific satiety on water perception).
(b) Choice of water during SSS compared to passive receipt of glucose before SSS (=influence of increased hedonic perception of water during SSS when compared to an inherently hedonic stimulus).
(c) Choice of water during SSS compared to passive receipt of glucose during SSS (=influence of increased hedonic perception of water during SSS when compared to SSS reduced hedonic perception of glucose).
(d) Passive receipt of glucose before SSS compared to passive receipt of water before SSS (=comparison of glucose processing before SSS with a neutral stimulus).
(e) Passive receipt of glucose during SSS compared to passive receipt of water before SSS (=comparison of SSS reduced hedonic perception of glucose to a neutral stimulus).

*Second-level analysis.* Individual contrast images of participants were included in a random effects analysis. One-sample *t*-tests were performed to assess whole-brain group activation. Statistically significant effects were determined using a primary search criterion of *p* < 0.001 uncorrected, with a minimal cluster size of k > 30, and the results are reported at a significance threshold of *p* < 0.05 and corrected for multiple comparisons using family-wise error (FWE) correction.

*Correlational analyses.* Activation estimates from significant regions of interest were extracted using the MarsBaR toolbox ([39], RRID:SCR_009605). Spherical regions of interest (8 mm diameter) were centered around the coordinates of peak activation of significant clusters from the group analysis. Specifically, we created two spheres for the medial prefrontal cortex, based on the contrast Cue: choice of water during SSS compared to passive receipt of water before SSS and peak of activation x = 6, y = 44, and z = 20, as well as the contrast Infusion: choice of water during SSS compared to passive receipt of water before SSS and peak of activation x = 9, y = 59, and z = 8. Furthermore, we created a sphere for the lateral orbitofrontal cortex, based on the contrast Cue: choice of water during SSS compared to passive receipt of glucose during SSS and peak of activation x = −45, y = 47, and z = −7. Mean percent signal change was extracted separately for each participant from the respective contrasts of interest and was then subsequently used in correlational analyses with BMI and craving scores (Pearson product-moment correlation coefficient, two-tailed, *p* < 0.05). In addition, we performed whole-brain regression analyses with BMI, everyday food craving, and changes in liking over time as individual covariates in separate models, applying the same significance thresholds as described above.

### 2.5. Behavioral Analysis

The liking ratings of liquids were analyzed using *R* [40]. To assess the effect of sensory-specific satiety on ratings, we employed two strategies. The first consisted of a repeated measures ANOVA with liquid type (water vs. glucose) and timepoint as within-subject factors to capture the gradual change in pleasantness over the course of the task. Because the primary aim of the study was to assess sensory-specific satiety rather than concentration-specific effects, ratings from the 10% and 20% glucose solutions were combined into a single “glucose” condition. Since water was administered more often than glucose during the experiment, only the first 17 infusions of water were used in the ANOVA in order to match the number of trials. Secondly, to assess changes in liking over time, we performed a linear regression analysis separately for water and glucose (using all ratings: 17 for glucose and 20 for water). Individual slopes of participants’ liking ratings, which represent the rate and direction of change of liking ratings over time, were used for correlational analyses with brain activation, BMI, and psychometric assessments of eating styles and craving (Pearson product-moment correlation coefficient, two-tailed, *p* < 0.05).

## 3. Results

### 3.1. Behavioral Results: Liking Ratings Associated with Sensory-Specific Satiety

In our repeated measures ANOVA of liking ratings, we found a significant interaction between liquid type and timepoint (*F*_(16,304)_ = 6.2, *p* < 0.001, *η*^2^ = 0.25). Post hoc tests revealed a significant main effect of liquid type *F*_(1,19)_ = 26.32, *p* < 0.001, and *η*^2^ = 0.58 but no significant main effect of time (*p* = 0.077). Furthermore, we found that liking ratings of water increased over time (F_(1,15)_ = 11.17, *p* = 0.004, *R*^2^ = 0.427, with a regression coefficient (slope) of *β*_1_ = 0.067) and liking ratings of glucose decreased over time ((F_(1,15)_ = 31.33, *p* < 0.001, *R*^2^ = 0.676, *β*_1_ = −0.116; see Figure 1C). Consistent with the expected shift in hedonic preference, participants exclusively selected water on all choice trials in the second half of the task, demonstrating that the expected shift in hedonic preference was achieved.

### 3.2. BOLD Responses Associated with Sensory-Specific Satiety

#### 3.2.1. Taste Anticipation Phase

**Figure 2:**
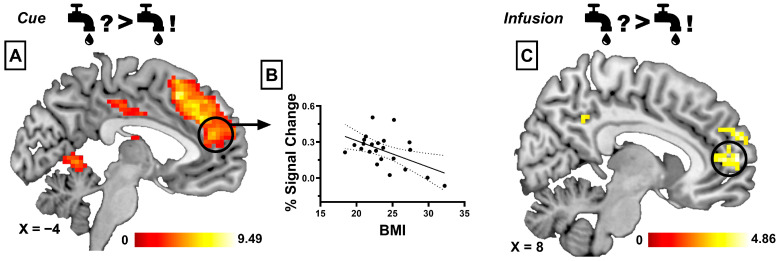
(**A**) Increased activation in the medial prefrontal cortex (marked with a black circle) when anticipating the taste of water during SSS compared to anticipating the taste of water before SSS. (**B**) Negative correlation between percent signal change extracted from the medial prefrontal cortex and individual BMI (*r* = −0.527, *p* = 0.009). (**C**) Increased activation in the medial prefrontal cortex (marked with a black circle) when comparing the taste of water during glucose-specific SSS to the taste of water before glucose-specific SSS. Color blobs indicate significant activation after family-wise error correction at the cluster level, overlaid on a standard MNI template for visualization.

**Figure 3:**
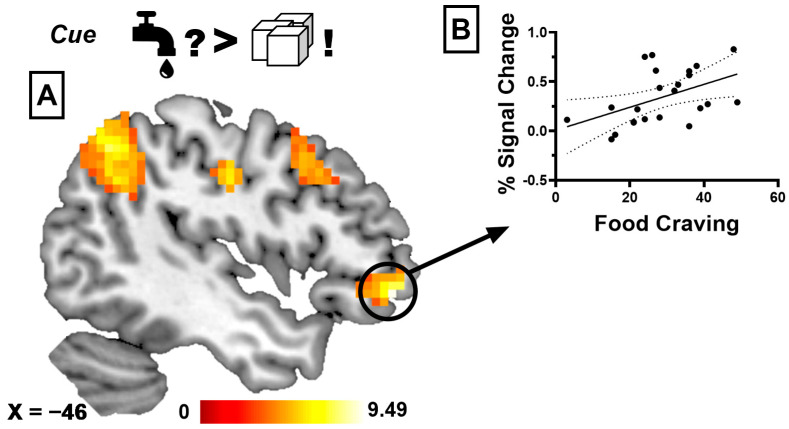
(**A**) Increased activation in the left lateral orbitofrontal cortex (marked with a black circle) when anticipating the taste of water during SSS compared to anticipating the taste of glucose during SSS. (**B**) Positive correlation between percent signal change extracted from the left lateral orbitofrontal cortex and state-dependent food craving ratings (*r* = 0.474, *p* = 0.022). Color blobs indicate significant activation after family-wise error correction at the cluster level, overlaid on a standard MNI template for visualization.

When anticipating the taste of water during SSS compared to anticipating the taste of water before glucose-specific SSS, we found that participants showed increased brain activation in the medial and anterior cingulate cortex (ACC), medial prefrontal cortex (mPFC), bilateral angular gyrus, anterior insula, and lateral orbitofrontal cortex (lOFC) and caudate nucleus (see Figure 2A). When anticipating the taste of water during SSS compared to anticipating the taste of glucose before SSS, we observed increased activation in the ACC, superior frontal cortex, middle frontal gyrus, and bilateral angular gyrus, as well as the inferior parietal lobule. Anticipating the taste of water compared to anticipating glucose during glucose-specific SSS revealed increased activation in the ACC, middle prefrontal cortex, lOFC, bilateral angular gyrus and postcentral gyrus, inferior parietal lobule, and middle frontal gyrus (see Figure 3A). When comparing the anticipation of glucose with water before glucose-specific SSS, we found increased activation in the superior temporal gyrus, middle occipital gyrus, and precuneus. Finally, when anticipating the taste of glucose during SSS to anticipating the taste of water before SSS, we observed increased activation in the superior temporal gyrus, middle occipital gyrus, precuneus, and parahippocampal gyrus. Table 2 gives a detailed description of the BOLD results.

#### 3.2.2. Infusion Phase

When comparing the taste of water during SSS to the taste of water before SSS, we observed a significant activation in the mPFC (see Figure 2C). The taste of water during SSS, compared to the taste of glucose before SSS, revealed increased activation in the mPFC and posterior cingulate cortex. In contrast to the anticipation phase, we did not observe significant activation when comparing the taste of glucose with the taste of water before SSS, nor when comparing the taste of water with the taste of glucose during SSS. However, we found increased activation in the dorsal medial cingulate cortex and posterior insula for the opposite contrast (taste of glucose compared to taste of water during SSS). Finally, the taste of glucose during glucose-specific SSS compared to water before SSS revealed significant activation in the mPFC. Table 3 gives a detailed description of the BOLD results.

#### 3.2.3. Correlational Analyses

We did not observe a significant correlation between slopes of liking ratings and BMI (for both water and glucose, *p*s > 0.05) or between liking ratings and psychometric scales (eating styles and food craving, *p*s > 0.05). For the contrast “anticipation of water during SSS compared to anticipation of water before SSS”, we found that the percent signal change extracted from the mPFC correlated negatively with the individual BMI (*r* = −0.527, *p* = 0.009; see Figure 2B). Finally, we observed a significant positive correlation between state-dependent food craving and percent signal change extracted from the lOFC during the contrast “anticipation of water compared to anticipation of glucose during SSS” (*r* = 0.474, *p* = 0.022; see Figure 3B). Finally, none of the separate whole-brain regression analyses (with BMI, everyday food craving, or changes in liking as covariates) revealed significant clusters at the corrected threshold.

## 4. Discussion

In this study, we investigated neural processing during both the anticipation and taste of water and glucose during SSS. We found that SSS-induced changes in hedonic perception were associated with a network of brain regions involved in reward and taste processing. Furthermore, brain activation during the anticipation of water during SSS was related to individual BMI, liking ratings, and food craving. Our results are in line with previous studies investigating SSS, but they extend these findings to context-dependent changes in hedonic perception during SSS. Consistent with our a priori hypotheses, these findings support the notion that SSS dynamically modulates neural activity in regions implicated in taste valuation and reward processing and that individual differences in BMI and food craving contribute to this modulation. Given the exploratory design and relatively small sample, these findings should be interpreted as preliminary evidence that requires replication in larger cohorts.

As expected, we found that liking ratings for water significantly increased throughout the experiment, whereas the opposite pattern emerged for glucose ratings. Accordingly, in line with the concept of SSS, the repeated exposure to glucose led to a decrease in its hedonic value, while water became more appealing. It is commonly believed that this adaptive shift in preference may reflect homeostatic mechanisms in place to promote dietary variety and maintain nutritional balance [41]. Energy-dense stimuli, such as glucose, can trigger early satiety responses, while zero-energy stimuli, like water, may gain in appeal as a compensatory mechanism [42]. However, SSS is also closely tied to subjective food preferences; for example, highly craved food shows a reduced decline in appeal during satiety, which has been associated with overeating [43]. Since we only administered very small amounts of liquids (1 mL per trial), our results are in line with previous studies showing that SSS can be triggered independently of physiological satiety via mere repeated exposure to the same taste [41].

When comparing the anticipation of water before and during SSS, we observed activation in a network of brain regions associated with reward and taste processing [44]. Specifically, we observed increased activation in primary and secondary taste areas located in the insula and OFC, respectively [27]. Both regions are crucial not only for sensual perception but also for hedonic evaluation and reward-related taste processing [21,45,46]. Previous studies found that a decrease in the pleasantness of food during SSS is associated with a decrease in activation in orbitofrontal regions [4,24]. Our results show that a context-dependent increase in hedonic perception is related to an increase in activation in this region, which underlines the importance of the OFC in food reward processing. Furthermore, we observed activation in the ACC and mPFC, which have been found to be involved in monitoring reward-related outcomes and adapting behavioral responses based on the changing value of rewards [47]. Given that participants had to choose between receiving water or glucose, activity in these regions when choosing water may reflect monitoring of the expected rewarding value of water under SSS.

Furthermore, we found that an increased BMI was related to reduced activation in the mPFC when comparing the anticipation of water before and during SSS. Previous studies found that increased reward processing during exposure to visual food cues is related to increased risk for future weight gain, whereas actual overeating is related to reduced activation in these regions [48]. However, a decreased neural response in the mPFC to palatable food tastes has been associated with future weight gain [49] and reduced activation in prefrontal regions, such as the ACC or mPFC, which has been found during food-related decision making in participants with obesity. [50]. Furthermore, obesity is related to reduced influence of satiety on neuronal processing of food stimuli [51,52]. Accordingly, individuals with a higher BMI may engage in habitual eating behaviors that are less responsive to changes in the value of food rewards and to internal cues of satiety since the motivational value of food is not adequately processed [53,54]. Our results indicate that similar processes may be involved in SSS; food evaluation in individuals with a higher BMI may be less responsive to changes in food palatability or pleasantness over time, which may enable excessive eating. This finding aligns with our a priori hypothesis that individual differences in BMI would modulate neural responses to hedonic revaluation during SSS. Because the stability of brain–behavior correlations is limited in small samples, this association should be regarded as tentative and hypothesis-generating. Our results show that the failure of behavioral or interventional studies to find reduced SSS in individuals with a higher BMI may be due to the fact that, despite intact SSS-induced reduction in hedonic perception of high-caloric food, the hedonic evaluation of uneaten food during SSS might be impaired.

Interestingly, comparing the anticipation of water with the anticipation of glucose during SSS revealed similar activation patterns as the comparison of water before and after SSS. Specifically, we observed activations in regions such as the ACC, mPFC, and lateral OFC. This overlap suggests that, irrespective of stimulus type, similar neural pathways are involved during SSS. This is in line with studies assessing the influence of satiety on different food tastes, where activation in the medial and orbitofrontal cortex, insula, and striatum was equally impacted by satiation for pleasant as well as for aversive stimuli [55]. Furthermore, we also found that activity in the lateral OFC was positively related to state-dependent food cravings. The lateral OFC plays a central role in motivational food reward processing [56,57,58], as well as in the conscious inhibition of desire for high-caloric food [59,60]. Activation in this region is not only crucial for taste discrimination [61] but has also been identified as a neural correlate of SSS [4]. Previous studies observed a positive relation between activation in the lateral OFC and food craving during processing of high-caloric food cues [62] but a negative relation during the receipt (i.e., tasting) of high-caloric food stimuli [63]. Accordingly, our results underline the hypothesized central role of the lateral OFC in SSS and show that reward value representation in the OFC may be modulated via inter-individual differences in food craving. This supports our hypothesis that craving influences neural representation of reward value during SSS, although this finding should be interpreted with caution, given the small sample size.

Similar to the anticipation of water, the taste of water during SSS was associated with increased activation in the mPFC. This finding is consistent with behavioral studies showing that SSS has an equal impact on the “liking” and “wanting” of tastes [64,65,66], suggesting that the mPFC may track changes in hedonic value associated with both anticipatory as well as consummatory processing during SSS. This finding was predicted by our hypothesis that SSS-related increases in water pleasantness would be paralleled by increases in mPFC activation during both anticipation and tasting. However, tasting water during SSS was associated with deactivation in the posterior insula when compared to tasting glucose. The posterior insula has been identified as the location of the human primary gustatory cortex [20,61] and as a hedonic hotspot involved in processing sucrose taste [67]. Furthermore, this region is crucial to taste aversion learning [68], and deactivation in the posterior insula has been related to a reduction in drug-induced cravings [69,70,71]. Given that activation in this region was stronger for the “disliked” glucose taste, our results indicate that the shift in hedonic value associated with SSS may partly be mediated by increased aversive processing in the insula. Beyond these preliminary correlations, the primary contribution of this work lies in demonstrating the feasibility and sensitivity of a novel gustatory fMRI paradigm. This proof-of-concept study provides methodological groundwork for future preregistered studies with larger samples that can more robustly assess brain–behavior associations. Furthermore, the COVID-19 pandemic has highlighted how gustatory and satiety-related paradigms may be affected by broader public health contexts. Future research should, therefore, consider how study designs can be adapted to remain feasible and valid under such conditions; for example, by integrating pandemic-related factors, such as infection history or long-term sensory changes [72,73,74].

### Limitations

Several limitations must be considered that potentially reduce the generalization and impact of our findings. Given that large sample sizes are required to obtain reproducible brain–behavior correlations [75], the relatively small sample size of the current study must be taken into account when interpreting the findings. Our study was exploratory in nature, was not preregistered, and did not include a formal a priori power calculation. Although our sample size is comparable to previous fMRI work in this field, the relatively small number of participants combined with multiple contrasts substantially increases the risk of false positives. We mitigated this risk by applying stringent family-wise error correction at the group level and by providing explicit theoretical motivation for each contrast in the methods. Nevertheless, because our study employed an exploratory whole-brain design, and given that correlational findings are particularly sensitive to sample size, caution is necessary when generalizing to larger populations. Thus, the findings should be considered exploratory and hypothesis-generating, providing a basis for future confirmatory studies. Furthermore, although our participants had a wide range of BMIs (18.4–32.2), only one participant had a BMI above 30. Thus, the implications of our results for obesity are very limited, and caution is required when extrapolating to this group. Future studies should specifically investigate SSS in participants with obesity to determine the relevance of our findings for the development and maintenance of obesity. We investigated a mixed group of 11 hormonally active females and 12 males, but did not consider the menstrual cycle, which has been shown to influence neuronal food processing [76]. Due to the small sample size, an analysis of potential sex differences or menstrual cycle effects was not feasible, which limits the interpretability of sex-related findings. Moreover, we did not assess hormonal satiety signalling during SSS, and given the pivotal function of gastrointestinal peptides in the gut–brain axis [77], this represents a further limitation of our study. Although participants were instructed to fast for approximately twelve hours prior to the scan, including abstaining from black coffee or tea, we did not obtain biochemical confirmation of fasting status. This absence of objective verification represents a limitation, although the primary aim of the study was to assess changes in subjective liking rather than to induce fullness or physical satiety, and only very small amounts of liquids were administered as stimuli. Additionally, we chose plain water as a familiar zero-energy stimulus with a neutral baseline value, which allowed us to study context-dependent increases in pleasantness under sensory-specific satiety without introducing an artificial control solution. Nevertheless, we cannot fully exclude that thirst regulation or fluid balance contributed to the observed hedonic shifts, and future studies should more systematically assess hydration status and thirst perception to disentangle these effects. Because fixed glucose deliveries alternated predictably in concentration during the second half of the task, anticipatory responses may have been influenced by trial predictability. Although these effects were modeled in the cue regressors, future studies could randomize concentration order to minimize such effects. Finally, we did not assess taste sensitivity in our participants. Since increased SSS for sweet food has been observed in individuals with high sweet taste sensitivity [78], this may have influenced our results.

## 5. Conclusions

In the present study, we assessed the neurobiological basis of SSS-induced changes in hedonic perception during the expectation and receipt of water and glucose. We found that a broad network of reward- and taste-related brain regions is involved during SSS. Furthermore, we found that individuals with higher BMI experience altered reward sensitivity during SSS and that increased food craving was related to increased activation in regions associated with food reward processing. The OFC has been identified as central to SSS in previous studies, and our results extend this finding by showing that the medial PFC is an important region for tracking the reward value of concurrent food stimuli during SSS. While the observed activation patterns largely overlap with previous studies on the neural processing of food reward and SSS, the present findings extend this work by demonstrating that a neutral zero-energy stimulus can acquire hedonic value in a context-dependent manner. Although water may not be an optimal comparator in all paradigms, in the present context, it provided a neutral reference stimulus that enabled the detection of relative increases in pleasantness. Future studies should further refine the interpretation of hedonic revaluation in SSS by including non-nutritive sweeteners as comparison stimuli. Taken together, our results add to the existing evidence that the appeal of food is influenced not only by physiological satiety but also by sensory factors related to craving and reward systems in the brain. These findings should be regarded as preliminary, hypothesis-generating evidence that a neutral zero-energy stimulus can acquire hedonic value in a context-dependent manner. Future studies should include well-powered, multi-site, preregistered replication designs to confirm and extend these findings and to establish sex-specific research protocols that consider female hormonal status and menstrual cycle effects.

## Figures and Tables

**Table 1:** Participant characteristics.

	Mean ± SD
Female/Male	11/12 (*n* = 23)
Age	28.6 ± 10.1
BMI kg/m^2^	23.8 ± 3.3 (range 18.4–32.2)
FCQ-S	28.6 ± 11.1
FCQ-T	52.6 ± 19.2
DEBQ—Restraint Eating	1.9 ± 0.7
DEBQ—Emotional Eating	1.8 ± 0.6
DEBQ—External Eating	2.6 ± 0.7
MWT-B	30.1 ± 4

FCQ-S: Food Craving Questionnaire—State, FCQ-T: Food Craving Questionnaire—Trait, DEBQ: Dutch Eating Behavior Questionnaire, MWT-B: Vocabulary-Based Test for the Assessment of Premorbid Intelligence. DEBQ German population norms [36]: restrained eating M = 2.23 ± 0.87, emotional eating M = 1.70 ± 0.76, external eating M = 2.57 ± 0.74.

**Table 2:** Increases in BOLD response associated with the anticipation of water and glucose.

* Water During SSS > Water Before SSS.*						
Brain Regions	k	t-Value	*p*-Value	x	y	z
Anterior Cingulate Cortex/Medial Frontal Cortex	1741	9.48	<0.001	6	44	20
Right Angular Gyrus/Inferior Parietal Lobule	417	6.41	<0.001	51	−46	44
Left Angular Gyrus/Inferior Parietal Lobule	607	8.16	<0.001	−45	−58	47
Right Anterior Insula/Orbitofronal Cortex	129	7.62	<0.001	48	23	−7
Left Anterior Insula/Orbitofronal Cortex	236	6.37	<0.001	−45	20	−13
Right Middle Temporal Gyrus	169	7.35	<0.001	66	−31	−1
Left Middle Temporal Gyrus	186	7.14	<0.001	−57	−40	−4
Right Posterior Cerebelum (Crus 1)	188	5.79	<0.001	36	−73	−22
Left Posterior Cerebelum (Crus 1)	183	6.13	<0.001	−27	−73	−28
Anterior Vermis	74	5.81	0.002	0	−55	−1
Medial Cingulate Cortex	72	5.45	0.002	3	−19	38
Caudate Nucleus	39	5.22	0.042	15	−1	20
*** Water During SSS > Glucose Before SSS***						
** Brain Regions**	**k**	**t-value**	***p*** **-value**	**x**	**y**	**z**
Right Angular Gyrus/Inferior Parietal Lobule	72	5	0.003	48	−46	44
Left Angular Gyrus/Inferior Parietal Lobule	240	7	<0.001	−48	−49	44
Middle Frontal Gyrus	94	7.02	0.001	−36	17	50
Anterior Cingulate Cortex/Superior Frontal Cortex	313	6.18	<0.001	−9	23	38
Anterior Cingulate Cortex	40	6.08	0.043	9	47	5
*** Water During SSS > Glucose During SSS***						
** Brain Regions**	**k**	**t-value**	***p*** **-value**	**x**	**y**	**z**
Middle Prefrontal Cortex	155	7.39	<0.001	−36	14	53
Left Orbitofrontal Cortex	73	7.22	0.004	−45	47	−7
Right Angular Gyrus/Inferior Parietal Lobule	184	6.31	<0.001	54	−55	47
Left Angular Gyrus/Inferior Parietal Lobule	369	6.91	<0.001	−48	−49	44
Right Postcentral Gyrus	88	6.89	0.001	60	−7	32
Left Postcentral Gyrus	113	6.26	<0.001	−48	−13	38
Superior Frontal Cortex/Anterior Cingulate Cortex	792	6.75	<0.001	−9	20	53
Middle Frontal Gyrus	85	5.32	0.002	39	26	35
Posterior Cerebelum (Crus 1)	45	5.02	0.035	−27	−70	−25
*** Glucose Before SSS > Water Before SSS***						
** Brain Regions**	**k**	**t-value**	***p*** **-value**	**x**	**y**	**z**
Superior Temporal Gyrus	144	6	<0.001	60	−52	23
Middle Occipital Gyrus	36	6	0.041	−18	−91	−1
Precuneus	45	5	0.016	6	−55	47
*** Glucose During SSS > water Before SSS***						
** Brain Regions**	**k**	**t-value**	***p*** **-value**	**x**	**y**	**z**
Superior Temporal Gyrus	244	6.69	<0.001	51	−49	23
Middle Occipital Gyrus	58	4.88	0.011	−30	−85	29
ParaHippocampal Gyrus	47	4.87	0.028	30	−28	−19
Precuneus	70	4.54	0.005	−12	−49	44

SSS = sensory-specific satiety, k = cluster size (voxels). Whole-brain results significant at P_FWE_ < 0.05 cluster level are reported, with a cluster-defining threshold of *p* < 0.001 and an uncorrected and minimal cluster size of k > 30.

**Table 3:** Increases in BOLD response associated with the taste of water and glucose.

*Water During SSS > Water Before SSS*						
*Brain Regions*	k	t-Value	*p*-Value	x	y	z
Medial Prefrontal Cortex	169	4.85	<0.001	9	59	8
***Water During SSS > Glucose Before SSS***						
Medial Prefrontal Cortex	428	7	<0.001	12	50	8
Posterior Cingulate Cortex	115	6	0.001	6	−49	38
***Glucose During SSS > Water During SSS***						
Dorsal Medial Cingulate Cortex	135	6.56	0.001	6	11	47
Posterior Insula	113	4.86	0.003	45	−4	2
***Glucose During SSS > Water Before SSS***						
Medial Prefrontal Cortex	84	6	0.005	3	50	32

SSS = sensory-specific satiety, k = cluster size (voxels). Whole-brain results significant at P_FWE_ < 0.05 cluster level are reported, with a cluster-defining threshold of *p* < 0.001 and an uncorrected and minimal cluster size of k > 30.

## Data Availability

The authors will make the raw data supporting this article’s conclusions available upon request due to privacy reasons.
